# Cooling and Sterile Inflammation in an Oxygen-Glucose-Deprivation/Reperfusion Injury Model in BV-2 Microglia

**DOI:** 10.1155/2021/8906561

**Published:** 2021-11-05

**Authors:** Jana Lücht, Nele Rolfs, Sylvia J. Wowro, Felix Berger, Katharina R. L. Schmitt, Giang Tong

**Affiliations:** ^1^German Heart Centre Berlin, Department of Congenital Heart Disease/Pediatric Cardiology, Berlin, Germany; ^2^Berlin Institute of Health, Berlin, Germany; ^3^Charité-Universitätsmedizin Berlin, Department of Congenital Heart Disease/Pediatric Cardiology, Berlin, Germany

## Abstract

**Objective:**

Cold-inducible RNA-binding protein (CIRBP) has been shown to be involved not only in cooling-induced cellular protection but also as a mediator of sterile inflammation, a critical mechanism of the innate immune response in ischemia/reperfusion (I/R) injury. The role of microglia and its activation in cerebral I/R injury warrants further investigation as both detrimental and regenerative properties have been described. Therefore, we investigated the effects of cooling, specifically viability, activation, and release of damage associated molecular patterns (DAMPs) on oxygen glucose deprivation/reperfusion- (OGD/R-) induced injury in murine BV-2 microglial cells.

**Methods:**

Murine BV-2 microglial cells were exposed to 2 to 6 h OGD (0.2% O_2_ in glucose- and serum-free medium) followed by up to 19 h of reperfusion, simulated by restoration of oxygen (21% O_2_) and nutrients. Cells were maintained at either normothermia (37°C) or cooled to 33.5°C, 1 h after experimental start. Cultured supernatants were harvested after exposure to OGD for analysis of DAMP secretions, including high-mobility group box 1 (HMGB1), heat shock protein 70 (HSP70), and CIRBP, and cytotoxicity was assessed by lactate dehydrogenase releases after exposure to OGD and reperfusion. Intracellular cold-shock proteins CIRBP and RNA-binding motif 3 (RBM3) as well as caspases 9, 8, and 3 were also analyzed via Western blot analysis. Furthermore, inducible nitric oxide synthase (iNOS), ionized calcium-binding adaptor molecule 1 (Iba1), tumor necrosis factor-*α* (TNF-*α*), interleukin-6 (IL-6), interleukin-1*β* (IL-1*β*), interleukin-1*α* (IL-1*α*), monocyte chemotactic protein 1 (MCP-1), transforming growth factor *β* (TGF*β*), CIRBP, and RBM3 gene expressions were assessed via reverse transcription polymerase chain reaction, and TNF-*α*, IL-6, and IL-1*β* releases into the cultured supernatants were assessed via enzyme-linked immunosorbent assays (ELISA).

**Results:**

Prolonged exposure to OGD resulted in increased BV-2 necrotic cell death, which was attenuated by cooling. Cooling also significantly induced cold-shock proteins CIRBP and RBM3 gene expressions, with CIRBP expression more rapidly regulated than RBM3 and translatable to significantly increased protein expression. DAMPs including HMGB-1, HSP70, and CIRBP could be detected in cultured supernatants after 6 h of OGD with CIRBP release being significantly attenuated by cooling. Exposure to OGD suppressed cytokine gene expressions of IL-1*β*, TNF-*α*, MCP-1, and TGF*β* independently of temperature management, whereas cooling led to a significant increase in IL-1*α* gene expression after 6 h of OGD. In the reperfusion phase, TNF-*α* and MCP-1 gene expressions were increased, and cooling was associated with significantly lower TGF*β* gene expression. Interestingly, cooled Normoxia groups had significant upregulations of microglial activation marker, Iba1, IL-1*β*, and TNF-*α* gene expressions.

**Conclusion:**

BV-2 microglial cells undergo necrotic cell death resulting in DAMP release due to OGD/R-induced injury. Cooling conveyed neuroprotection in OGD/R-injury as observable in increased cell viability as well as induced gene expressions of cold shock proteins. As cooling alone resulted in both upregulation of microglial activation, expression of proinflammatory cytokines, and cold shock protein transcript and protein expression, temperature management might have ambiguous effects in sterile inflammation. However, cooling resulted in a significant decrease of extracellular CIRBP, which has recently been characterized as a novel DAMP and a potent initiator and mediator of inflammation.

## 1. Introduction

Ischemic brain injury resulting from a deprivation of oxygen and nutrients initiates multiple damage mechanisms including sterile inflammation. Due to necrotic cell death, damage-associated molecular patterns (DAMPs) are released into the extracellular matrix, which can activate microglial cells by binding to respective pattern recognition receptors, e.g., toll-like receptors (TLR) [1]. It has been shown that activation and proliferation of microglial cells in the ischemic region of the brain occur during the first 3 days after cerebral ischemia [2, 3]. As resident immune cells of the brain, microglial cells control the inflammatory process via release of cytokines and matrix metalloproteinases, leading to further recruitment of microglial cells and peripheral leukocyte and monocyte immigration due to a weakened blood brain barrier [4]. In addition to initiating sterile inflammation, microglial cells also clear cellular detritus via phagocytosis and contribute to neuronal regeneration after ischemia/reperfusion (I/R) injury [5]. Several studies have shown that microglial cell activation leads to a detrimental effect in I/R injury [6–8], whilst others demonstrate a neuroprotective effect [9–11]. Therefore, the role of microglial cells in I/R injury warrants further investigation.

Cooling or targeted temperature management (TTM) is an established neuroprotective strategy for I/R injury in the brain routinely applied in neonates after perinatal hypoxic-ischemic encephalopathy and in adults after cardiac arrest [12–15]. A meta-analysis of *in vivo* studies analyzing the effect of hypothermia on focal cerebral ischemia showed that cooling decreased infarct size significantly by 44% and resulted in improved functional outcome. Reduction of infarct size was dependent on time of initiation and degree of cooling. Although cooling to ≤31°C initiated before or at the beginning of ischemia showed the most effective reduction of infarct size, mild cooling to 35°C was also shown to be beneficial [16]. Moreover, a recent meta-analysis focusing on preclinical studies from 2010 to 2015 confirmed the previously described effects of cooling and suggested endovascular cooling as a neuroprotective method even if initiated during ischemia [17]. In clinical trials, intravascular cooling administered by cold saline infusions has been shown to be feasible in patients suffering from ischemic stroke [18, 19]. However, in the intravascular cooling in the treatment of stroke 2 (ICTuS 2) trial, patient recruitment was stopped as thrombectomy, proven to be an efficient treatment for the selected group of patients, and was not included as a treatment. Due to small sample sizes, no statistically significant differences were reported. Yet, patients treated with intravascular cooling presented increased incidence of pneumonia [19]. This is consistent with the results of another randomized multicenter trial analyzing mild cooling (34.0–35.0°C) for 12–24 h, achieved either by intravenous infusion or surface cooling within 6 h of symptom onset to 90 minutes within the start of thrombolysis. Unfortunately, patients' outcomes could not be analyzed due to lack of fundings [20].

Cooling has been shown to be involved in multiple protective mechanisms in the setting of ischemic stroke. Besides attenuating excitotoxicity, calcium influx, oxidative stress, and neuronal apoptosis, there is growing evidence that cooling also reduces the inflammatory response after I/R injury in the brain [21]. Although a well-established protective mechanism lies within the general reduction of metabolism, cold shock proteins such as cold-inducible RNA binding protein (CIRBP) and RNA-binding motif 3 (RBM3) are upregulated under cooling and convey neuroprotection [22–24]. However, extracellular CIRBP has been shown to act as a DAMP and a potent modulator of inflammation. In addition to being released upon necrotic cell death, CIRBP can also be secreted upon, e.g., hypoxia. Administration of recombinant CIRBP resulted both *in vitro* and *in vivo* in increased levels of proinflammatory cytokines and the release of other DAMPs [25]. Moreover, patients suffering from hemorrhagic shock and sepsis showed increased peripheral blood levels of CIRBP, which correlated with poor outcome [25, 26]. Concordantly to other DAMPs such as high-mobility group box 1 (HMGB1), CIRBP binds to the toll-like receptor 4 (TLR4) and myeloid differentiation factor 2 (MD2) complex to initiate inflammation [25]. Furthermore, recent *in vitro* studies show that CIRBP also binds to triggering receptor expressed on myeloid cells-1 (TREM-1) and interleukin 6 receptor (IL-6 R) to activate another pathway of proinflammatory response or promote macrophage endotoxin tolerance [27, 28]. Thus, recent experimental and clinical data underline that extracellular CIRBP is a potent initiator and modulator of inflammation.

The aim of this study is to investigate the effects of cooling initiated during oxygen glucose deprivation (OGD) as a potential neuroprotective strategy in murine BV-2 microglial cells. Therefore, we analyzed the impact of cooling on OGD-induced necrotic cell death, DAMPs release, and cytokines as well as cold-shock protein expressions.

## 2. Materials and Methods

### 2.1. Cell Culture

Immortalized murine BV-2 microglial cells [29] were kindly provided by Prof. Ullrich (Zurich, Switzerland). BV-2 cells were cultured as previously described in high-glucose Dulbecco's modified Eagle's medium (DMEM) supplemented with 1% natrium-pyruvate (Biochrom), 10% heat inactivated fetal bovine serum (FBS, Biochrom), and incubated at 37°C, 21% O_2,_ and 5% CO_2_ [30]. Both cultivating media as well as experimental media were supplemented with 100 U/ml penicillin and 100 *μ*g/ml streptomycin (Merck Milipore).

### 2.2. Simulation of Ischemia/Reperfusion Injury

As previously described by our group, deprivation of oxygen and glucose was used to simulate ischemia. Briefly, cells were incubated in glucose/serum-free DMEM at 0.2% O_2_ and 5% CO_2_ in a CO_2_ incubator (Binder, Tuttlingen, Germany). Reperfusion was simulated by restoration of nutrients and incubation at 21% O_2_. Control groups were kept in DMEM supplemented with glucose and 10% FBS for the duration of the experiment.

### 2.3. Time-Temperature Protocol

**Figure 1:**
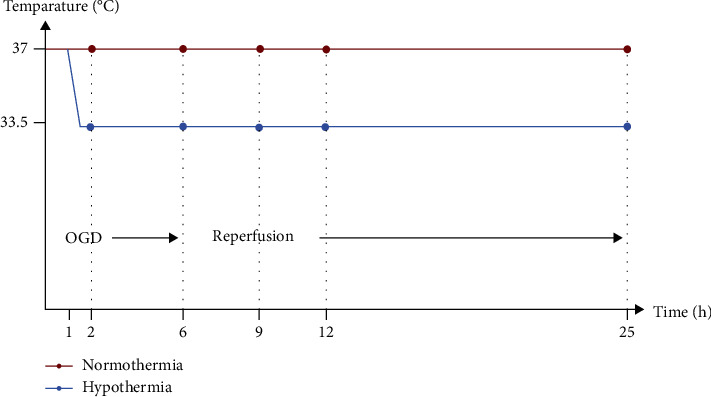
Time-temperature protocol. Cells were exposed to 2 or 6 h of oxygen-glucose deprivation (OGD, 0.2% O_2_ in glucose/serum-free medium) followed by up to 19 h of reperfusion (21% O_2_ in medium containing glucose and serum) and incubated at 37°C or cooled 1 h after experimental start to 33.5°C. Samples were collected directly after 2 or 6 h of OGD and after 3 h, 6 h, and 19 h of reperfusion, respectively.

Prior to experimental start, 500,000 cells were seeded in a 21 cm^2^ petri dish (Sarstedt) and maintained for 24 h. Cells were exposed to OGD and reperfusion according to a time-temperature protocol as illustrated in Figure 1. Experimental groups were exposed to OGD for 2 or 6 h, followed by up to 19 h of reperfusion. Cooling (33.5°C) was initiated after 1 h of OGD and continued until the end of the experiment. Control groups were incubated at 37°C or 33.5°C, 21% O_2_, and 5% CO_2_ for the duration of the experiment.

### 2.4. Assessment of Necrotic Cell Death via Lactat Dehydrogenase (LDH) Assay

Necrotic cell death was analyzed via LDH release into cultured supernatants at each experimental timepoints using a colorimetric Cytotoxicity Detection Kit (Roche Diagnostics) according to manufacturer's instructions. The extinction was measured at 490 nm minus 630 nm using a microtiter plate reader (Thermo Fisher Multiskan Ascent). Cytotoxicity is presented as a percentage in relation to maximum LDH content assessed by a lysed normoxic control group as previously described by our research group [30].

### 2.5. Protein Isolation and Western Blot Analysis

At each experimental time point, cells were harvested, and supernatants were collected for the isolation of intracellular and extracellular proteins, respectively. For intracellular protein analysis, cells were centrifuged at 6,000 x g for 10 minutes, and cell pellets were lysed in radio-immuno precipitation assay (RIPA) buffer supplemented with protease and phosphatase inhibitors (1 : 100, Sigma-Aldrich). Protein concentration was assessed via Pierce Bicinchoninic Acid (BCA) Protein Assay (Thermo Scientific). Extracellular proteins were precipitated using a trichloroacetic acid protocol as previously described [30]. Both intra- and extracellular protein samples were incubated with Pierce Lane Marker Reducing Sample Buffer (Thermo Scientific) at 95°C for 5 min and loaded onto a 15% sodium dodecyl sulfate (SDS) polyacrylamide gel for electrophoresis. Afterwards, proteins were transferred onto a polyvinylidene fluoride membrane (PALL Life Sciences) overnight at 30 V using a tank blotting procedure (Bio-Rad Laboratories). Membranes were then blocked for 1 h at room temperature with 5% BSA (Carl Roth) for *β*-Actin, Caspase 1, Caspase 8, Heat shock protein 70 (Hsp70), and HMGB1 or 5% dry milk (Applied Biosystems) for CIRBP, RBM3, Caspase 3, and Caspase 9 in TBS+0.1% Tween 20. Primary antibodies for *β*-Actin (1 : 20000, Cell Signaling, Cat#4967), Caspase 1 (1 : 1000, Cell Signaling, Cat#67314), Caspase 8 (1 : 1000, Cell Signaling, Cat#9429), Caspase 9 (1 : 1000, Cell Signaling, Cat#9508), Caspase 3 (1 : 1000, Cell Signaling, Cat#9662), Hsp70 (1 : 1000, Cell Signaling Technology, Cat#4872) HMGB1 (1 : 2000, Chondrex, Cat#7028), CIRBP (1 : 1000, Abclonal, Cat#A6080), and RBM3 (1 : 1000, Proteintech, Cat#14363-1-AP) were diluted in blocking solution and incubated overnight at 4°C. Secondary antibodies (anti-rabbit IgG-HRP, Dianova) were incubated for 1 h at room temperature (1 : 10,000 for CIRBP and HMGB1, otherwise 1 : 20,000). Dura Super Signal West (Thermo Fisher Scientific) was used to visualize protein expression, captured using a ChemiDoc™ Imaging Systems, and Image Lab™ Software (Bio-Rad) was used for densitometry analysis.

### 2.6. Enzyme-Linked Immunosorbent Assay (ELISA)

Secreted tumor necrosis factor-*α* (TNF-*α*), interleukin-6 (IL-6), interleukin-1*β* (IL-1*β*), TNF-*α*, IL-6, and IL-1*β* concentrations in cultured supernatants were analyzed at 6 h OGD and 12 h OGD and reperfusion (OGD/R) via ELISA (DuoSet Mouse, R&D Systems) in 96-well plates prepared according to manufacturer's instructions. Briefly, captured antibody-precoated plates were incubated with blocking solution for 1 h at room temperature, supernatants were collected and transferred in duplicates onto a 96-well-plate and incubated overnight at 4°C, and detection antibodies were transferred at a dilution 1 : 60 and incubated for 3 h, followed by light-protected incubation with HRP-conjugated streptavidin for 20 minutes at room temperature. Extinction was measured at 450 nm and 540 nm using a microtiter plate reader (Thermo Fisher Multiskan Ascent).

### 2.7. RNA Isolation and Reverse Transcription Polymerase Chain Reaction (RT-PCR)

Total RNA from BV-2 cells was isolated via acidic phenol/chloroform extraction using RNA Pure™ (Peqlab) followed by DNA digestion using a Turbo DNA-free™ Kit (Ambion) according to manufacturer's instructions. RNA concentration and purity were assessed by spectrophotometric measurements at 260 nm and 280 nm with a Nanodrop 2000 (Nanodrop) and agarose gel electrophoresis. Reverse transcription was performed using 1 *μ*g total RNA via a High Capacity cDNA Reverse Transcription Kit (Applied Biosystems) in a thermal cycler (PTC200, MJ Research) according to manufacturer's instructions. Expression of target genes and GAPDH as reference keeping control was analyzed by real-time qPCR using the TaqMan Gene Expression Assays (summarized in Table 1) and StepOnePlus™ Real-Time PCR System (Applied Biosystems) according to manufacturer's recommendations. We assessed relative quantification of gene expression normalized to glycerinaldehyd-3-phosphat dehydrogenase (GAPDH) as reference gene via the ∆∆−*C*_*t*_ method, and results are depicted as fold changes [31].

### 2.8. Statistical Analysis

Data was analyzed and illustrated using GraphPad Prism 9 (GraphPad Software, Inc., La Jolla, CA, USA). Groups were compared using one-way analysis of variance (ANOVA) with Tukey posttest. Data from at least 3 independent experiments are presented as mean ± standard deviation (SD), and *p* values < 0.05 were considered significant.

## 3. Results

### 3.1. Necrotic Cell Death

**Figure 2:**
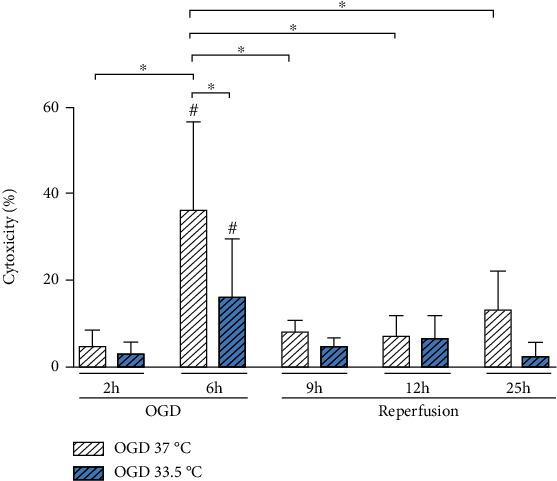
Necrotic cell death measured by LDH release, depicted as % cytotoxicity relative to positive (100%) and Normoxia (0%) controls. Cells were exposed to 2 and 6 h of oxygen-glucose deprivation (OGD, 0.2% O_2_ in glucose/serum-free medium) followed up to 19 h of reperfusion (21% O_2_ in medium containing glucose and serum) and incubated at 37°C or cooled 1 h after experimental start to 33.5°C. Data from at least 3 individual experiments presented as mean ± SD. Statistical analysis was conducted using one-way analysis of variance (ANOVA) with Tukey posttest; ^∗^*p* < 0.05 for group comparison and ^#^*p* < 0.05 for comparison to Normoxia 37°C were considered significant.

As necrotic cell death due to ischemic brain injury leads to the release of DAMPs and the initiation of sterile inflammation, we compared 2 h versus 6 h duration of OGD in order to establish a time-temperature protocol for induced injury in the BV-2 microglia. Cytotoxicity was assessed via LDH release in the cultured supernatants as illustrated in Figure 2. Exposure to OGD at 37°C for 2 h did not result in significant BV-2 cytotoxicity, whereas 6 h of OGD led to a significant increase in LDH release relative to Normoxia 37°C control. Cooling to 33.5°C initiated after 1 h OGD effectively attenuated BV-2 cell death at the end of the 6 h OGD phase. Restoration of nutrients and oxygen to 21% in the simulated reperfusion phase did not result in further increased cell death. In fact, % cytotoxicity was significantly lower at all investigated reperfusion time points (9, 12, and 25 h) relative to 6 h OGD at 37°C and also not significantly higher than Normoxia 37°C control. Maintenance of cooling at 33.5°C during the reperfusion phase resulted in lower observable cytotoxicity, but did not reach significancy.

### 3.2. Apoptotic Cell Death

**Figure 3:**
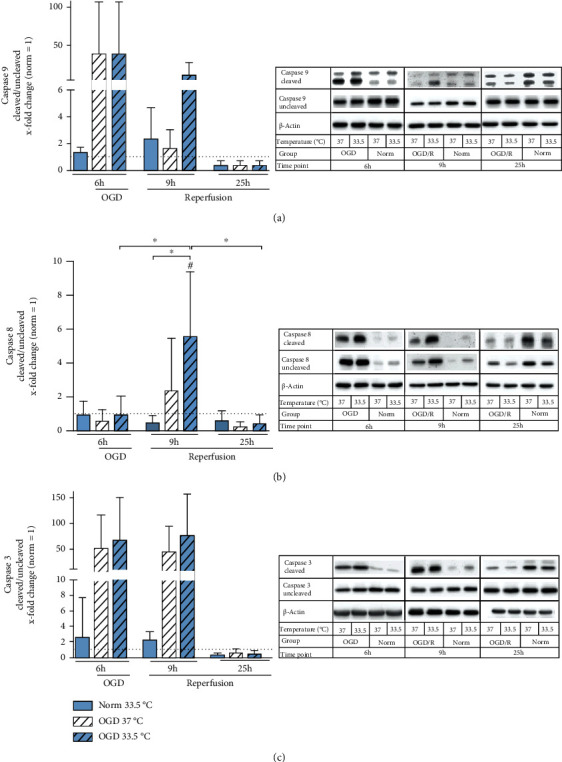
Western blot analysis of apoptotic cell death as assessed by cleavage of (a) caspase 9 as initiator of intrinsic apoptosis, (b) caspase 8 as initiator of extrinsic apoptosis, and (c) caspase 3 as effector caspase presented as *x*-fold change relative to Normoxia 37°C control and representative immunoblots at respective timepoints. Cells were exposed to 6 h of oxygen-glucose deprivation (OGD, 0.2% O_2_ in glucose/serum-free medium) followed by 3 h and 19 h of reperfusion (21% O_2_ in medium containing glucose and serum) and incubated at 37°C or cooled 1 h after experimental start to 33.5°C. Data from at least 3 individual experiments are presented as mean ± SD. Statistical analysis was conducted using one-way analysis of variance (ANOVA) with Tukey posttest; ^∗^*p* < 0.05 for group comparison and ^#^*p* < 0.05 for comparison to Normoxia 37°C were considered significant. Panels show representative immunoblots.

To fully understand the effect of exposure of BV-2 microglia to OGD/R and cooling on programmed apoptotic cell death, we investigated both the intrinsic and extrinsic apoptotic pathways by assessing activation of initiator caspases 9 and caspase 8, as well as their common effector caspase 3 (Figure 3). Activation of caspase 9, the initiator caspase of intrinsic apoptosis, was highly observable after 6 h of OGD in both cooled and normothermic cells but not significant relative to Normoxia 37°C control. Furthermore, activation of caspase 9 steadily decreased after reperfusion (Figure 3(a)). Extrinsic initiator caspase 8 was significantly activated after reperfusion (9 h after experimental start) in cells subjected to cooling and OGD/R relative to Normoxia 37°C control, as well as to Normoxia group cooled to 33.5°C (Figure 3(b)). In consistency with the observable activation of the initiator caspases, effector caspase 3 was also observed to be activated both during OGD and early reperfusion (9 h), though not to significancy and no influence by temperature management was observed (Figure 3(c)).

### 3.3. Oxidative Stress and Microglial Activity

**Figure 4:**
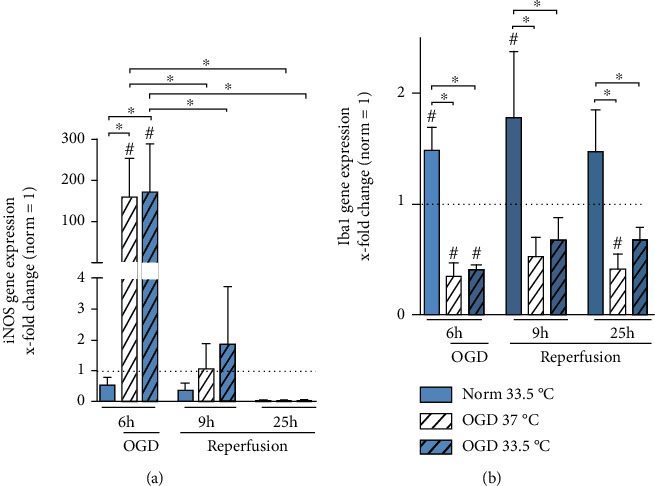
RT-qPCR analyzing (a) iNOS and (b) Iba1 gene expression presented as *x*-fold change relative to Normoxia 37°C control at respective timepoints. Cells were exposed to 6 h of oxygen-glucose deprivation (OGD, 0.2% O_2_ in glucose/serum-free medium) followed by 6 h and 19 h of reperfusion (21% O_2_ in medium containing glucose and serum) and incubated at 37°C or cooled 1 h after experimental start to 33.5°C. Data from at least 3 individual experiments are presented as mean ± SD. Statistical analysis was conducted using one-way analysis of variance (ANOVA) with Tukey posttest; ^∗^*p* < 0.05 for group comparison and ^#^*p* < 0.05 for comparison to Normoxia 37°C were considered significant.

We analyzed inducible nitric oxide synthase (iNOS) gene expression as an indicator for oxidative stress. iNOS gene expression was significantly increased after 6 h of OGD in both cooled and normothermic groups. iNOS gene expression was significantly higher after 6 h exposure to OGD relative to Normoxia 37°C control and decreased significantly after reperfusion (12 h and 25 h) as compared to 6 h OGD (Figure 4(a)). Cooling had no significant effect on iNOS transcript during exposure to OGD/R. Ionized calcium-binding adaptor molecule 1 (Iba1) is specifically expressed in microglial cells and is commonly used as a marker for microglia activation [32]. Exposure to OGD/R significantly suppressed Iba1 gene expression in comparison to Normoxia 37°C control, and cooling had no observable effect (Figure 4(b)). However, cooling to 33.5°C under normoxic conditions led to significant upregulation of Iba1 transcripts (6 h and 12 h) relative to normothermic Normoxia control as well as OGD/R groups.

### 3.4. Cold Shock Proteins

**Figure 5:**
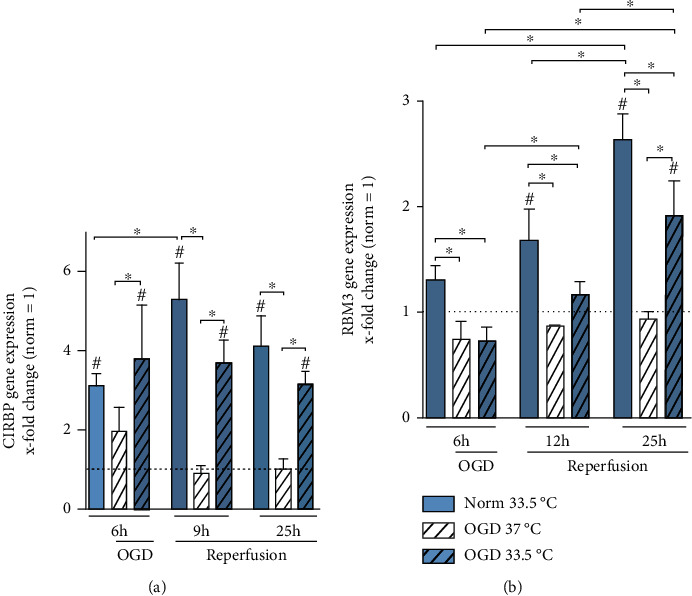
RT-qPCR analyzing cold shock proteins (a) CIRBP and (b) RBM3 gene expression presented as *x*-fold change relative to Normoxia 37°C control at respective timepoints. Cells were exposed to 6 h of oxygen-glucose deprivation (OGD, 0.2% O_2_ in glucose/serum-free medium) followed by 6 h and 19 h of reperfusion (21% O_2_ in medium containing glucose and serum) and incubated at 37°C or cooled 1 h after experimental start to 33.5°C. Data from at least 3 individual experiments are presented as mean ± SD. Statistical analysis was conducted using one-way analysis of variance (ANOVA) with Tukey posttest; ^∗^*p* < 0.05 for group comparison and ^#^*p* < 0.05 for comparison to Normoxia 37°C were considered significant.

**Figure 6:**
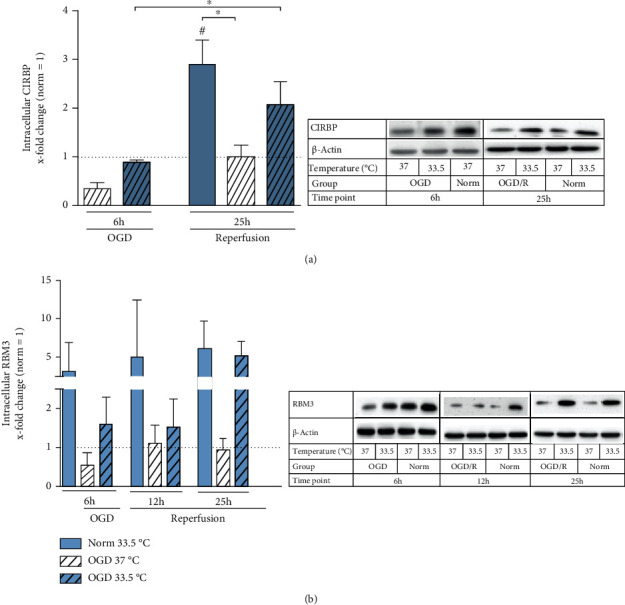
Western blot analysis analyzing intracellular cold shock proteins (a) CIRBP and (b) RBM3 presented as *x*-fold change relative to Normoxia 37°C control and representative immunoblots at respective timepoints. Cells were exposed to 6 h of oxygen-glucose deprivation (OGD, 0.2% O_2_ in glucose/serum-free medium) followed by 6 h and 19 h of reperfusion (21% O_2_ in medium containing glucose and serum) and incubated at 37°C or cooled 1 h after experimental start to 33.5°C. Data from at least 3 individual experiments are presented as mean ± SD. Statistical analysis was conducted using one-way analysis of variance (ANOVA) with Tukey posttest; ^∗^*p* < 0.05 for group comparison and ^#^*p* <0.05 for comparison to Normoxia 37°C were considered significant. Panels show representative immunoblots.

A family of cold shock protein has been observed to be induced under hypothermia and other stress conditions. Therefore, we analyzed both gene expression as well as intracellular protein expression of cold shock proteins RBM3 and CIRBP. CIRBP transcript was significantly upregulated by cooling in all experimental groups, regardless of exposure to Normoxia or OGD/R, at all observable time points (Figure 5(a)). Cooling-induced RBM3 gene expression kinetics was observably slower than that of CIRBP and reached significancy after 12 h in the Normoxia group and 25 h in the OGD/R group (Figure 5(b)). Moreover, cooling-induced RBM3 expression significantly increased with prolonged duration of cooling, observable by the significantly highest expression at 25 h in both Normoxia and OGD/R conditions. Interestingly, intracellular CIRBP protein expression was not as dynamically induced under cooling and was only significantly higher than Normoxia 37°C control after 25 h cooling under normoxic condition (Figure 6(a)). We observed a tendency towards higher RBM3 protein expression under cooling for both Normoxia and OGD/R treatment conditions, which did not reach significancy (Figure 6(b)).

### 3.5. Release of DAMPs and Cold-Shock Proteins

**Figure 7:**
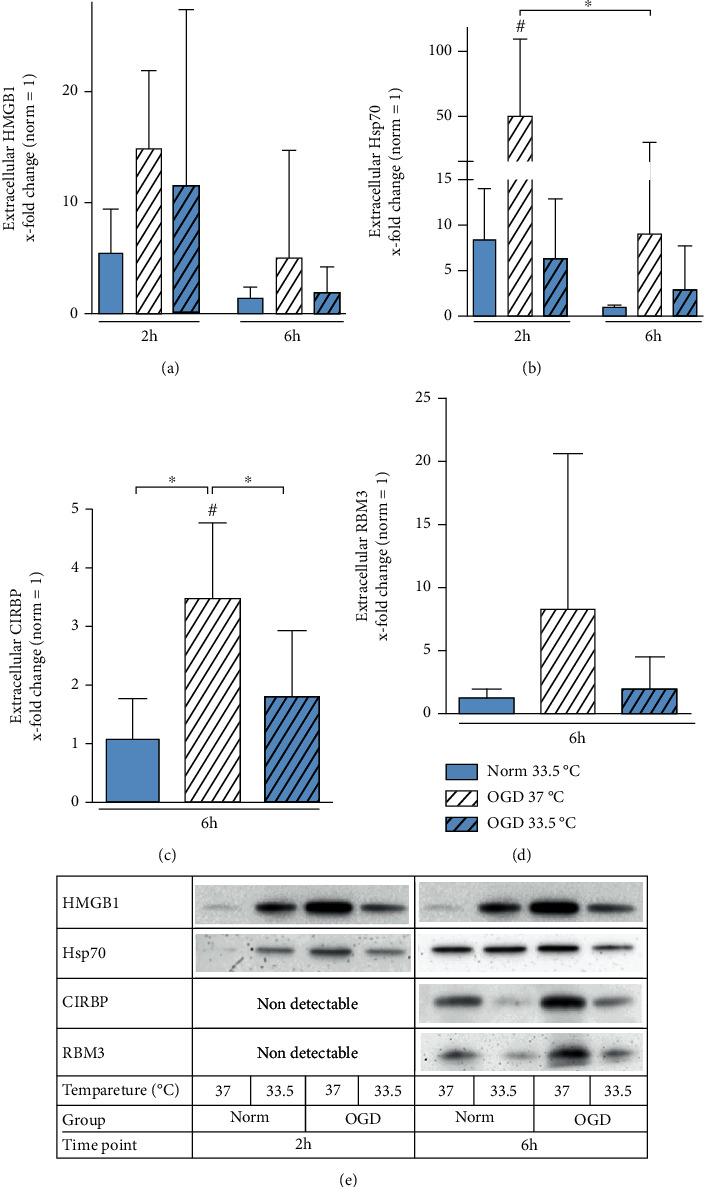
Western blot analysis analyzing extracellular proteins and DAMPs. (a) HMGB1, (b) Hsp70, (c) CIRBP, and (d) RBM3 presented as *x*-fold change relative to Normoxia 37°C control and (e) representative immunoblots at respective timepoints. Cells were exposed to 2 h and 6 h of oxygen-glucose deprivation (OGD, 0.2% O_2_ in glucose/serum-free medium) and incubated at 37°C or cooled 1 h after experimental start to 33.5°C. Data from at least 3 individual experiments are presented as mean ± SD. Statistical analysis was conducted using one-way analysis of variance (ANOVA) with Tukey posttest; ^∗^*p* < 0.05 for group comparison and ^#^*p* < 0.05 for comparison to Normoxia 37°C were considered significant. Panels show representative Immunoblots.

Necrotic cell death has been observed to lead to the release of DAMPs, which can initiate the innate inflammatory response. Therefore, we investigated the release of HMGB-1, HSP70, and CIRBP into cultured supernatants after 2 and 6 h of OGD to evaluate the contribution of microglia to DAMPs release. We observed the greatest release of HMGB1 after 2 h exposure to OGD, which did not reach significancy (Figure 7(a)). HSP70 release was significantly higher from cells subjected to OGD for 2 h than Normoxia 37°C control and OGD exposure for 6 h (Figure 7(b)). Neither CIRBP nor RBM3 were detectable in the culture supernatants after 2 h of OGD. However, CIRBP release was significantly higher after 6 h of OGD relative to Normoxia 37°C control and could be attenuated by cooling (Figure 7(c)). RBM3 release showed a higher tendency after 6 h exposure to OGD but did not reach significancy (Figure 7(d)).

### 3.6. Cytokine Gene Expressions and Releases

**Figure 8:**
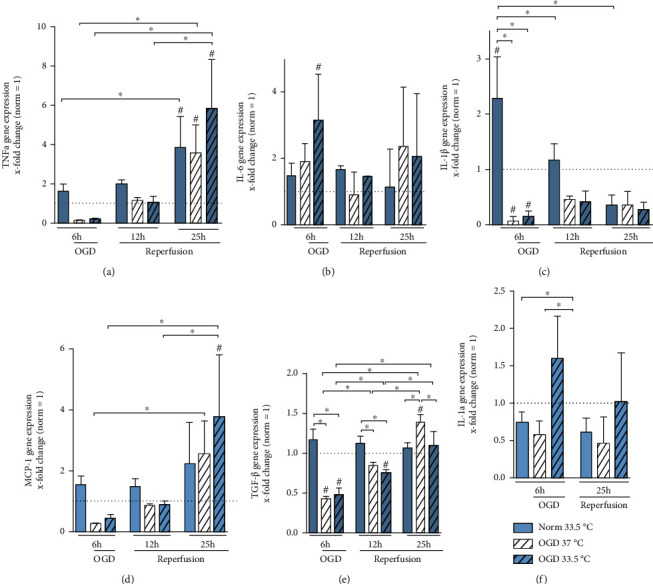
RT-qPCR analyzing cytokine gene expression. (a) TNF-*α*, (b) IL-6, (c) IL-1*β*, (d) MCP-1, (e) TGF*β*, and (f) IL-1*α* presented as *x*-fold change relative to Normoxia 37°C control at respective timepoints. Cells were exposed to 6 h of oxygen-glucose deprivation (OGD, 0.2% O_2_ in glucose/serum-free medium) followed by 6 h and 19 h of reperfusion (21% O_2_ in medium containing glucose and serum) and incubated at 37°C or cooled 1 h after experimental start to 33.5°C. Data from at least 3 individual experiments are presented as mean ± SD. Statistical analysis was conducted using one-way analysis of variance (ANOVA) with Tukey posttest; ^∗^*p* < 0.05 for group comparison and ^#^*p* < 0.05 for comparison to Normoxia 37°C were considered significant.

In order to assess the inflammatory response in BV-2 microglia, we analyzed a panel of cytokine gene expressions, including TNF-*α*, IL-6, IL-1*β*, monocyte chemotactic protein 1 (MCP-1), transforming growth factor *β* (TGF-*β*), and interleukin-1*α* (IL-1*α*) (Figures 8(a)–8(f), respectively). Interestingly, exposure to 6 h OGD generally resulted in suppressed cytokine expressions with observable significancy in IL-1*β* and TGF-*β* expressions (Figures 8(c) and 8(e)). Only IL-6 expression was observed to be unaffected by OGD and even significantly higher expressed relative to Normoxia 37°C control in the 6 h cooled OGD group (Figure 8(b)). Cooling alone significantly induced IL-1*β* expression under normoxic conditions (Figure 8(c)). Both TNF-*α* and MCP-1 showed a similar expression pattern to IL-1*β* with a tendency towards suppression by OGD. However, TNF-*α* gene expression was significantly induced in all investigated groups after 25 h, and MCP-1 was significantly upregulated in cooled cells relative to Normoxia 37°C control after reperfusion (25 h OGD/R), in contrast to IL-1*β*, which remained downregulated during reperfusion (Figures 8(a), 8(c), and 8(d)). IL-6 and IL-1*α* showed a similar expression pattern in the 6 h OGD phase, with IL-6 significantly upregulated relative to Normoxia 37°C control and IL-1*α* significantly upregulated relative to cooled Normoxia and noncooled OGD-treated groups. Similarly, we did not detect any significant regulation of IL-6 and IL-1*α* during reperfusion (Figures 8(b) and 8(f)). Furthermore, we analyzed anti-inflammatory TGF-*β* gene expressions, which was attenuated by OGD and remained suppressed after 12 h in cooled OGD/R group. Interestingly, TGF-*β* expression recovered after 25 h of OGD/R and was significantly increased in the uncooled cells treated with OGD/R relative to Normoxia 37°C control as well as both cooled Normoxia- and OGD/R-treated groups (Figure 8(e)).

**Figure 9:**
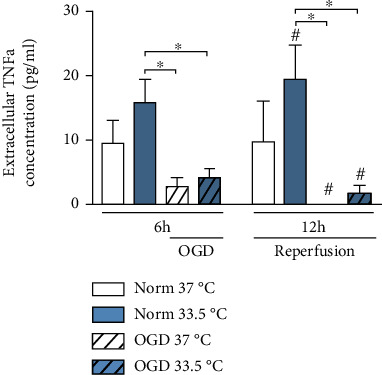
ELISA analysis of TNF-*α* release presented as absolute concentration. Cells were exposed to 6 h of oxygen-glucose deprivation (OGD, 0.2% O_2_ in glucose/serum-free medium) followed by 6 h of reperfusion (21% O2 in medium containing glucose and serum). Cells were incubated at 37°C or cooled 1 h after experimental start to 33.5°C. Data from at least 3 individual experiments presented as mean ± SD. Statistical analysis was conducted using one-way analysis of variance (ANOVA) with Tukey posttest; ^∗^*p* < 0.05 for group comparison and ^#^*p* < 0.05 for comparison to Normoxia 37°C were considered significant.

Next, we analyzed the release of proinflammatory cytokines into supernatants via ELISA. BV-2 cell exposure to OGD did not result in a significant increase in TNF-*α* release (Figure 9). On the contrary, exposure to 6 h of OGD resulted in a significant decrease in TNF-*α* release relative to the cooled Normoxia-treated cells. Attenuation of TNF-*α* release by OGD continued into the reperfusion phase (12 h OGD/R) where both cooled and noncooled OGD/R-treated groups were significantly lower relative to both cooled and noncooled Normoxia-treated groups. Additionally, IL-6 and IL-1*β* were both nondetectable in the cultured supernatants for all experimental groups.

## 4. Discussion

Sterile inflammation is an important component of I/R injury in the brain where resident immune microglial cells are activated within the first hours after ischemia [2, 3]. Microglia have been shown to contribute to both inflammatory and regenerative responses after I/R injury, thus providing an interesting target for potential neuroprotective therapies [1].

Cooling during and after ischemic brain injury has been shown to influence microglial activation and cytokine release [33–36]. Therefore, the role of microglial activation in ischemic brain injury remains a prevailing research topic. While microglia activation has been observed *in vivo* to be associated with a significant decrease in neurogenesis after focal ischemia and their specific inhibition resulted in increased neurogenesis in the hippocampus, other studies have reported that the number of activated microglia negatively correlate with ischemic damage [2, 8]. Therefore, we investigated the effect of cooling as an established neuroprotective strategy and the role of sterile inflammation on OGD/R-induced injury in murine BV-2 microglial cells.

Our simulated I/R injury model resulted in significant increase in necrotic BV-2 cell death after 6 h exposure to OGD, which could be attenuated by cooling to 33.5°C (Figure 2). Moreover, we also observed increased apoptotic programmed cell death (Figure 3) as seen in caspase 3 activation in the microglia by exposure to OGD/R. Apoptosis during OGD was primarily via the intrinsic caspase 9 pathway, which was not influenced by temperature management, and via the extrinsic caspase 8 pathway during reperfusion. Interestingly, extrinsic caspase 8 activation was significantly upregulated in the cooled cells as compared to both control groups. We also observed a significant suppression in Iba1 expression in the microglia by exposure to OGD/R. Iba1 is an intracellular protein that is specifically expressed in microglial cells, and its upregulation has been used as a marker for microglial activation [32]. Furthermore, Iba1 has been shown to play an important role in phagocytosis [37, 38] and is upregulated *in vivo* by exposure to ischemia [39]. Selective hypothermia therapy in the brain, however, has been reported to attenuate microglial activation as seen in reduced Iba1 gene expression [40]. Contrary to these findings, Iba1 was significantly upregulated in our cooled Normoxia groups. However, we did observe significant decreases in Iba1 gene expression after 6 h of OGD that could not be restored by cooling (Figure 4(b)). Furthermore, 6 h of exposure to OGD resulted in significantly decreased cell viability, which may also influence the degree of observed microglial activation. Our finding corresponds with a previous *in vivo* study reporting that short durations of ischemia are associated with activation of local microglia, whereas longer duration of ischemia resulted in their degeneration [2].

Moreover, iNOS gene expression was significantly increased from exposure to OGD. Normally, iNOS is undetectable in resting microglia but is upregulated by ischemia, traumatic brain injury, or inflammation, which leads to NO production and oxidative stress [41–43]. iNOS has been shown to be detrimental in ischemic brain injury as experiments with iNOS knockout mice have shown significantly reduced infarct areas and pharmacological inhibition of iNOS results in less ischemic brain damage [44, 45]. Cooling to 33°C has been shown to reduce iNOS expression and NO production in both an *in vivo* model of focal brain ischemia and a neuroinflammatory model induced by lipopolysaccharide (LPS) injection [46]. Furthermore, cooling to 33.5°C has been reported to decrease iNOS gene and protein expression in LPS-stimulated BV-2 cells [47]. However, we did not observe any significant attenuation by cooling of the upregulated iNOS gene expression in the noncooled cells after 6 h of OGD, but we did observe significant decreases in iNOS in both groups after reperfusion (Figure 4(a)).

To further assess the effect of OGD/R on BV-2 microglia activation, we investigated the gene expressions of proinflammatory cytokines (IL-1*α*, IL-6, TNF-*α*, and IL-1*β*), anti-inflammatory cytokine TGF*β*, and chemotactic cytokine MCP-1. TNF-*α*, IL-1*β*, MCP-1, and TGF*β* gene expressions were all significantly suppressed by exposure to OGD in the BV-2 microglia (Figures 8(a) and 8(c)–8(e)). Zhou et al. report an iNOS-dependent upregulation of TNF-*α* in BV-2 cells subjected to hypoxia [48]. As we detected decreased TNF-*α* gene expression after 6 h of OGD, no correlation between iNOS and TNF-*α* regulation could be established in our findings. Previous studies also show that cooling is accompanied by a reduction in TNF-*α*, IL-6, and IL-1*β* expressions in the setting of ischemic brain injury and neuroinflammation [33–36]. Seo et al. investigated the influence of cooling initiation time on cytokine expression and reported that TNF-*α*, IL-1*β*, iNOS, and IL-6 were all attenuated by cooling independent of time of initiation, but early initiation of cooling was most effective in reducing oxidative stress and transcription of proinflammatory cytokines [36]. Xiong et al. showed that postischemic hypothermia attenuates both TNF-*α* and IL-6 gene expressions but described a different expression kinetic where TNF-*α* peaked at 12 and 24 h and IL-6 peaked at 24 and 72 h after ischemia [35]. Since our experimental model investigated up to a maximum of 19 h after reperfusion, a difference in expression kinetics and potential effects of cooling at later timepoints could not assessed.

IL-1*α* and IL-1*β* are well-established proinflammatory cytokines that have been shown to convey detrimental effects in I/R injury in the brain [49]. Interestingly, we detected differing gene expression kinetics due to OGD in combination with cooling, where IL-1*α* was increased and IL-1*β* was significantly downregulated by cooling after 6 h of OGD (Figures 8(c) and 8(f), respectively). IL-1*α* has been reported to be released following necrotic cell death, whereupon functioning as a DAMP depending on its subcellular localization. Since we only investigated IL-1*α* gene expression, its subcellular localization and potential role in as a DAMP in sterile inflammation warrant further investigation.

In contrast to the IL-1 cytokine family, TNF-*α* and IL-6 have been shown to convey both detrimental and neuroprotective effects [50–52]. In classic IL-6 signaling, binding to the IL-6 membrane bound receptor is considered protective, whereas binding to its soluble receptor is considered proinflammatory [50]. As we only analyzed IL-6 gene expression, no concrete conclusion on potential effects of IL-6 regulation due to temperature management in OGD/R-injured BV-2 cells can be made. However, IL-6 gene expression in our model of OGD/R-injury in BV-2 microglial cells differs from the other analyzed cytokines as it shows a significant upregulation at 6 h of OGD due to cooling but otherwise no significant regulation due to OGD/R or temperature management (Figure 8(b)).

Additionally, we analyzed secretion levels of TNF-*α*, IL-6, and IL-1*β* by ELISAs. IL-6 and IL-1*β* were below assay detection limits; however, TNF-*α* secretion was downregulated relative to Normoxia control after exposure to OGD/R and independent of temperature management (Figure 9). Postischemic upregulation of TNF-*α* protein levels due to cooling has been reported in an *in vivo* model using postischemic cooling to 33°C [34]. We observed an upregulation of TNF-*α* gene expression due to both cooling alone and after 25 h of OGD/R (Figures 8(a) and 9). Similar to IL-6, TNF-*α* has also been described as a pleiotropic cytokine, dependent on its binding to either membrane bound or soluble receptor [53]. *In vivo* studies have shown that TNF deficiency results in increased infarction volumes and behavioral dysfunction [51, 52]. Here, we report significant regulations of pro- and anti-inflammatory, as well as pleiotropic cytokines expressions in BV-2 microglia due to exposure to OGD/R and cooling.

Several studies have reported upregulations of CIRBP and RBM3 expressions by hypoxia [54–56]. Liu et al. showed *in vivo* that CIRBP gene expression was significantly upregulated in the cortex after 24 h of ischemia, where cooling as well as the combined treatment of ischemia with cooling resulted in increased CIRBP gene expression 6 h after initiation [55]. Furthermore, Zhou et al. reported upregulation of both gene and protein expressions of CIRBP *in vivo*, as well as in BV-2 cells subjected to 20 h up to 48 h of ischemia/hypoxia [56]. We also observed significant increases in RBM3 and CIRBP gene expressions, as well as their intracellular protein expressions in OGD/R-induced injured BV-2 cells treated with cooling (Figures 5 and 6). Moreover, cooling alone also resulted in significant upregulations of both cold-shock protein gene expressions (Figure 5). As most of the past *in vitro* and *in vivo* studies have analyzed cold shock protein expressions after several hours up to days of ischemia or hypoxia exposure, the lack of observable induced cold shock protein expressions due to OGD exposure alone in our study may be attributed to a shorter duration of OGD.

Interestingly, CIRBP gene expression showed a rapid upregulation after 6 h of OGD at 33.5°C and stayed significantly upregulated through all investigated timepoints, whereas RBM3 was significantly upregulated only after 19 h of reperfusion (OGD/R) at 33.5°C (Figure 5). This is in correlation with our previously reported findings of different expression kinetics between CIRBP and RBM3 in organotypic hippocampal slice cultures (OHSC) treated with moderate hypothermia (33.5°C), where CIRBP gene expression was significantly increased after only 4 h of cooling, while RBM3 showed a delayed regulation with an increase after 24 h of cooling [57], thus confirming the difference in expression kinetics between CIRBP and RBM3 in a BV-2 microglial monoculture. This has also been reported outside of the brain where increased CIRBP transcripts were observed after 24 h of cooling to 25°C, compared to 5 days of cooling required for RBM3 induction in human lung fibroblasts [58]. This difference in the dynamics of cold-shock protein regulation may play an important role in the mechanism of neuroprotection induced by cooling. RBM3 has been shown to reduce neuronal apoptosis in ischemic brain injury [24, 59, 60]. Apoptosis is an active and, therefore, energy-dependent cell death mechanism that primarily contributes to reperfusion-induced injury. Si et al. provide promising data that RBM3 is a key player in the formation of stress granules after OGD-induced injury, a cellular rescue mechanism prohibiting apoptosis [60, 61]. Overexpression of RBM3 in PC12 cells resulted in attenuated apoptotic cell death and increased cell viability, whereas RBM3 knockdown had the opposite effect [60]. Another recent study investigating RBM3 knockout in mice after ischemic brain injury focusing on neural stem/progenitor cells (NSPC) shows that RBM3 plays an important role in neuronal regeneration after ischemic brain injury [59]. In summary, RBM3 has been reported to convey neuroprotection on multimodal levels with studies focusing on intracellular effects. However, homologous cold-shock protein CIRBP is known to have both neuroprotective as well as detrimental effects depending on its location. It has been shown that upregulation of intracellular CIRBP increases cell viability in neural stem cells, which is abolished via small interfering RNA (siRNA) knockdown of CIRBP [23]. Furthermore, overexpression of CIRBP restored cell proliferation in neural stem cells treated with hypoxia, indicating a regulating function of CIRBP in the cell cycle [62].

In our study, cooling alone without OGD/R resulted in an increase in gene expressions of Iba1, cold-shock proteins CIRBP and RBM3, and TNF-*α*, MCP-1, IL-1*β*, and TGF*β* (Figures 4(b), 5(a), 5(b), 6(a), 6(b), 8(a), and 8(c)–8(e), respectively). Our results indicate that cooling, whilst increasing cell viability and expression of cold shock proteins in a model of OGD/R-induced injury, also activates BV-2 microglial cells and is accompanied by an upregulation of both pro- and anti-inflammatory cytokine gene expressions and the release of TNF-*α*. Moreover, we have previously reported similar results in BV-2 microglial cells with Iba-1 and MCP-1 gene expressions being upregulated after 24 h of cooling [30]. Furthermore, TNF-*α* release was increased due to cooling alone in the reperfusion phase 12 h after experimental start (Figure 9). Thus, suggesting that cooling alone induces gene expressions of the microglial activation marker, Iba1, and proinflammatory cytokines. To our knowledge, there are no other studies investigating the potential activation of microglial cells by cooling. Because our study focused predominantly on transcriptional regulation by cooling, further investigation are needed in order to verify the potential proinflammatory effects of temperature management in the absence of OGD/R-induced injury.

To further investigate the role sterile inflammation in the setting of ischemic brain injury, we analyzed DAMP release from BV-2 microglia after OGD-induced injury (Figure 7). Although overexpression of HSP70 has been shown to be neuroprotective, extracellular HSP70 has been characterized as a DAMP that binds to a TLR and activates the inflammatory response [63–65]. High-mobility group 1 (HMGB1) protein is located in the nucleus and can be secreted by both monocytes and macrophages, as well as release after necrotic but not apoptotic cell death [66, 67]. HMGB1 is a well-characterized DAMP in the setting of stroke, where its *in vivo* neutralization results in decreased microglial activation, cytokine and iNOS expressions, and reduced permeability of the blood brain barrier [67, 68]. Clinical studies have shown that elevated HMGB1 serum levels could be detected in patients suffering from myocardial or cerebral ischemia [69]. *In vivo*, ischemic brain injury leads to a decrease of HMGB1 immunoreactive cells in the ischemic cortex and an increase of HMGB1 in serum, which could be attenuated by moderate cooling during ischemia [70].

OGD-induced necrotic microglia cell death resulted in the release of DAMPs, including HMGB1, HSP70, and CIRBP, into the cell cultured media (Figure 7). CIRBP release was significantly attenuated by cooling, but no attenuation in the release of HSP70 and HMGB1 by cooling was observable. However, we could observe a difference in the release dynamics of HMGB1 and HSP70 relative to CIRBP (Figures 7(a)–7(c), respectively). HSP70 and HMGB1 releases were highest after 2 h of OGD and decreased after 6 h, whereas extracellular CIRBP and RBM3 were only detectable after 6 h. Extracellular CIRBP has been identified as a novel DAMP acting as a potent mediator of inflammation, and RBM3 release was also investigated due to its high homology with CIRBP (Figure 7(d)). We observed a significant attenuation of CIRBP release by cooling, but only nonsignificant reduction in the release of HMGB1, HSP70, and RBM3. The detrimental effect of CIRBP in ischemic brain injury has been described by Zhou et al., as infarct volume could be attenuated by 61% in CIRBP knockdown mice 30 h after middle cerebral artery occlusion. The group also reports a translocation of CIRBP from the nucleus to the cytoplasm upon exposure to hypoxia in BV-2 microglial cells, followed by its eventual release after 20 h to 30 h of hypoxia [56]. In an *in vivo* model of deep hypothermic cardiac arrest, knockdown of CIRBP resulted in decreased cerebral injury and neuronal cell death [71]. Qiang et al. showed that CIRBP is released actively via lysosomal secretion in macrophages treated with hypoxia [25]. Concordantly to previous reports investigating the role of CIRBP in macrophages, CIRBP release appears to be an initiator of proinflammatory cascades involving microglial activation and cytokine release [25, 56, 71] and has been shown to be released earlier than TNF-*α* in ischemic brain injury models [56, 71]. While treatment of BV-2 cells with recombinant CIRBP induced TNF-*α* release via NF-*κ*B pathway, this was effectively abolished by CIRBP blockage [56]. It has been reported that incubation with supernatants from BV-2 cells treated with hypoxia induces apoptosis in neurons, which was reproduced by stimulating with recombinant CIRBP and reversed by blocking CIRBP via antiserum or siRNA [56, 71]. However, neurons may be more susceptible to extracellular CIRBP in terms of apoptosis induction. To our knowledge, this is the first report showing that both CIRBP and RBM3 are being released into extracellular matrix in OGD-induced injury BV-2 microglial cells. Whereas extracellular CIRBP promotes sterile inflammation and neuronal cell death, to our knowledge, there are no data on the deleterious effects of extracellular RBM3.

In the present study, we investigated the effects of cooling on BV-2 microglia subjected to OGD/R. In context of analyzing the role of temperature management on sterile inflammation in ischemic brain injury, it is important to note our study limitations. A microglial monoculture was used, so the contributions of other cerebral cells exposed to cooling- and OGD/R-induced injury cannot be assessed. Potential pleiotropic effects of the analyzed cytokines cannot be assessed strictly from the regulated gene transcription data presented. Lastly, our results indicate the passive release of CIRBP from necrotic BV-2 microglia. However, potential active release mechanisms of cold shock proteins, different release kinetics relative to HMGB1 and HSP70, and their role in sterile inflammation remains to be elucidated and warrants further research.

## 5. Conclusion

Cooling applied during OGD significantly attenuated necrotic cell death in BV-2 microglial cells. Cold-shock proteins RBM3 and CIRBP, both known to convey neuroprotection by increasing cell viability, inhibiting apoptosis, and promoting regenerative mechanisms after ischemic brain injury, were induced by cooling, but show different expression kinetics with a delay for RBM3. Exposure to OGD resulted in significantly higher CIRBP release that could be attenuated by intra-OGD cooling and correlated with cytotoxicity. As extracellular CIRBP is a potent and novel inductor and mediator of inflammation, our findings further support cooling as a potential neuroprotective strategy against sterile inflammation. Our study focused on and showed the potential protective effects of cooling in OGD/R-injured BV-2 microglial cells, but further investigation on the impact of extracellular cold-shock proteins and the innate immune response are needed.

## Figures and Tables

**Table 1:** List of RT-qPCR genes and assay IDs.

Gene	Assay ID
CIRBP	00483336_g1
GAPDH	99999915_g1
Iba1	00479862_g1
IL-1*α*	00439620_m1
IL-1*β*	00434228_m1
IL-6	00446190_m1
iNOS (iNOS-2)	00440502_m1
MCP-1 (Ccl-2)	00441242_m1
RBM3	01609819_g1
TGF*β*	01178820_m1
TNF-*α*	00443260_g1

## Data Availability

The raw data used to support the findings of this study are available from the corresponding author upon request.
